# Clinical and imaging features of intraosseous arteriovenous malformations in jaws: a 15-year experience of single centre

**DOI:** 10.1038/s41598-020-68967-3

**Published:** 2020-07-21

**Authors:** Xiao Li, Lixin Su, Deming Wang, Zhipeng Gui, Mengda Jiang, Xitao Yang, Yifeng Han, Liming Zhang, Lianzhou Zheng, Xindong Fan

**Affiliations:** 1 Department of Interventional Therapy, Shanghai Ninth People’s Hospital, Shanghai Jiao Tong University School of Medicine, No. 639 Zhi Zao Ju Rd., Shanghai, 200011 Shanghai People’s Republic of China; 2 Department of Oral and Cranio-Maxillofacial Surgery, Shanghai Ninth People’s Hospital, Shanghai Jiao Tong University School of Medicine, No. 639 Zhi Zao Ju Rd., Shanghai, 200011 Shanghai People’s Republic of China; 3 Department of Radiology, Shanghai Ninth People’s Hospital, Shanghai Jiao Tong University School of Medicine, No. 639 Zhi Zao Ju Rd., Shanghai, 200011 Shanghai People’s Republic of China

**Keywords:** Peripheral vascular disease, Oral cancer

## Abstract

Intraosseous arteriovenous malformations in jaws (j-AVMs) are rare congenital high-flow vascular anomalies with a high tendency of life-threatening haemorrhage and are regarded as one of the most dangerous haemorrhagic diseases in maxillofacial region. Pre-treatment clinical and imaging evaluations serve as the most important diagnostic modalities. A retrospective study involved 211 patients with j-AVMs from November 2003 to November 2017 was performed. The male-to-female ratio of j-AVMs was approximately 4:3. The mean age of the patients with j-AVMs is 21.86. Bleeding was the main complaint associated with j-AVMs. J-AVMs occurred in the mandible more often than in the maxilla (64.93% and 32.23%, respectively). Most j-AVMs (95.26%) occurred in the posterior teeth region. Classical imaging features of j-AVMs included: an unclear maxillary sinus with a mild ground-glass appearance (maxillary j-AVMs) and a clear oval or irregular lucency that is mostly centred on the root of the first molar (mandibular j-AVMs) on OPGs, enhancement in the cancellous bone on contrast-enhanced CTs. Other atypical features of j-AVMs were also concluded. A comprehensive diagnose system based on clinical and imaging features of j-AVMs could provide valuable reference data for clinical management of j-AVMs and help avoid improper iatrogenic trauma or delayed treatment.

## Introduction

AVMs are rare congenital high-flow vascular anomalies with an estimated prevalence of approximately 1/100,000 person-years^1,2^, and intraosseous arteriovenous malformations in jaws (j-AVMs) are rarer and associated with a high tendency of life-threatening haemorrhage either spontaneously or after oral surgeries^3^. The etiopathogenesis of j-AVMs remains unclear; however, it has been speculated that the lesions are the result of an embryologic abnormality in differentiation during the early stages of embryogenesis^4–7^. Due to the complex morphology of jaws and the existence of teeth and pulp arteries, j-AVMs are more difficult to identify and manage than other intraosseous AVMs.

However, due to the low prevalence of j-AVMs, a clinician might see one patient every few years, and it is extremely difficult to gain the experience necessary to diagnose such patients^8–10^. Historically, the terms “central haemangioma”, “arteriovenous aneurysm”, “cavernous haemangioma”, “pulsatile haemangioma”, and “angioma” of the jaw were used to describe this type of disease, which might cause confusion and misunderstanding of j-AVMs. In fact, closely related to the oral cavity and teeth, j-AVMs have precise anatomical and physiological characteristics that differ from AVMs in other parts of the body. A misunderstanding of j-AVMs may lead to incorrect diagnosis and management, resulting in serious iatrogenic injuries, even severe bleeding or death. Developing a comprehensive understanding of the clinical and imaging features of j-AVMs is highly important and could serve as the cornerstone for the proper management of j-AVMs.

There are few studies reporting its clinical characteristics with large sample sizes, except for a literature review of 50 reported mandibular AVMs^11^. Original studies investigating j-AVMs mostly involve separate case reports or series^12,13^. A previous study involving a series of twelve patients demonstrated that the clinical and radiological features were variable and might be confused with cystic lesions or tumours in the jaw^13^. However, reported cases are limited and unsystematically reviewed. Clinicians have been unable to fully understand the clinical and radiological features of j-AVMs in large samples.

Given the challenging diagnostic and management considerations for j-AVMs, this study was designed to retrospectively evaluate a large cohort of patients with j-AVMs at a single centre over a 15-year period to gain a better understanding of the spectrum of j-AVMs, including their demographic features, presentation signs and symptoms, and manifestations on orthopantomograms (OPGs) and contrast-enhanced computed tomography (CT).

## Results

### Demographics

**Figure 1:**
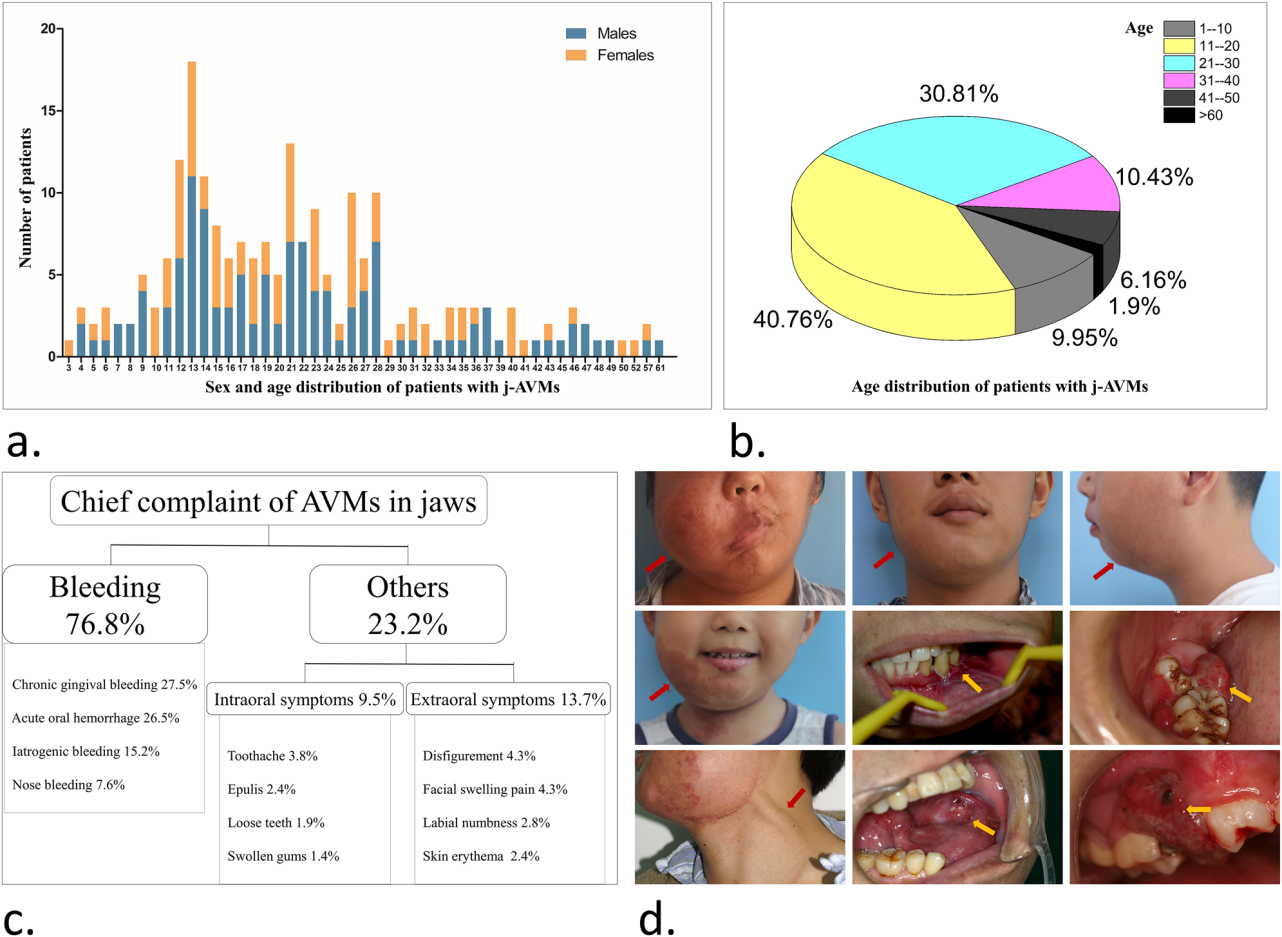
Demographic and clinical features of j-AVMs. (**a**,**b**) Age and sex distributions of the patients with j-AVMs. (**c**) Chief complaint in patients with j-AVMs. Acute oral haemorrhage refers to spontaneous circumstances or haemorrhage related to daily activities, such as chewing or brushing, instead of iatrogenic or second to trauma haemorrhage. (**d**) Extra-oral and intra-oral clinical manifestations of j-AVMs.

The study group consisted of 211 patients with a male-to-female ratio of approximately 4:3 (120 males, 91 females). The age at the first appointment ranged from 3–61 years with a mean of 21.86 years and a median of 20 years. The histograms of the age and sex distributions show that j-AVMs manifested differently according to age with the following two periods of high prevalence: there was a single peak at the age of 13 years and a plateau stage from the ages of 21–28 (Fig. 1a). Most j-AVMs (40.76%) manifested in patients aged 11–20 years (Fig. 1b).

### Presenting signs and symptoms

The presenting signs and symptoms included bleeding and other symptoms. Bleeding was the main complaint in the patients with j-AVMs and was present in 76.8% of all patients. The following four types of bleeding were observed in the study: chronic gingival bleeding, acute oral haemorrhage (which refers to spontaneous circumstances or related to daily activities, such as chewing or brushing, instead of iatrogenic causes or second to trauma), iatrogenic bleeding and nose bleeding. Chronic gingival bleeding and acute oral haemorrhage were the most common complaints and occurred in 27.5% and 26.5% of all patients, respectively. Iatrogenic bleeding (15.2%) was due to inappropriate surgical operations, such as the extraction of involved teeth, biopsy surgery and partial excision of the jaws. Additionally, 7.6% of the patients complained of nose bleeding. The other symptoms were divided into intra-oral and extra-oral symptoms. The intra-oral symptoms (9.5%) included toothache (3.8%), epulis (localized mass in the gingiva) (2.4%), loose teeth (1.9%) and swollen gums (1.4%). The extra-oral symptoms (13.7%) included disfigurement (4.3%), facial swelling pain (4.3%), labial numbness (2.8%) and skin erythema (2.4%) (Fig. 1c). The presenting signs and symptoms are shown (Fig. 1d).

### Anatomic distribution

The locations of j-AVMs were categorized as being in the maxilla/mandible/both and on the left/right/both sides. Certain anatomic regions were used to specify the involved location as follows: posterior teeth region (premolars and molars involved), anterior teeth region (incisors and canines involved) and other regions (other parts of the jaws in addition to the alveolar process). The results showed that AVMs occurred in the mandible more often than in the maxilla at percentages of 64.93% and 32.23%, respectively. Six of the 211 (2.84%) patients suffered from j-AVMs involving both the maxilla and mandible. Of these 211 cases, more j-AVMs were located on the left side (52.13%) than the right side (40.76%), and 7.11% of the patients suffered from bilateral j-AVMs. Most (95.26%) j-AVMs were classified as being in the posterior teeth region, specifically around the roots of the first molars. In total, 1.90% (4/211) of the j-AVMs occurred in the anterior teeth region, and 2.84% (6/211) of the j-AVMs occurred in other regions, including the mandibular symphysis, palatal suture, mental foramen, ascending ramus of the mandible, and palatal vault regions (Table 1).

**Table 1:** Anatomic Distribution of Arteriovenous Malformations in the Jaws.

Anatomic distribution	Number of patients	Percentage
Maxilla	68	32.23%
Mandible	137	64.93%
Maxilla and mandible	6	2.84%
Left	110	52.13%
Right	86	40.76%
Bilateral	15	7.11%
Anterior	4	1.90%
Posterior	201	95.26%
Other	6	2.84%
Total	211	100.00%

### OPG manifestations

**Figure 2:**
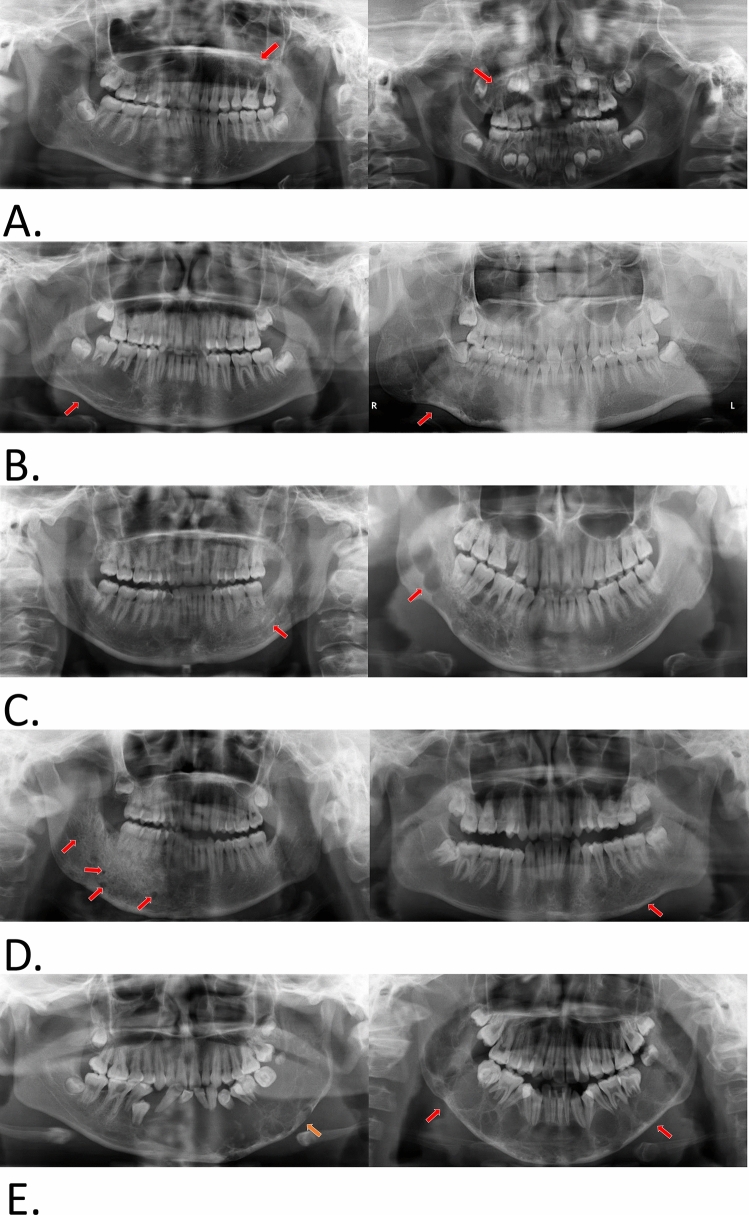
Orthopantomogram manifestations of j-AVMs in the maxilla (**a**) and mandible (**b**–**e**). (**a**) Unclear maxillary sinus and mild ground-glass appearance with involvement of the region around the roots of incisor (left) and first molar (right). The arrow indicates unclear maxillary sinus. (**b**) A clear oval or irregular hypodense shadow is mostly centred on the root of the first molar with a long axis along the mandibular canal and occasionally with root resorption. (**c**) Enlarged and wider mandibular canal than that on the contralateral side, and the mandibular canal was replaced by an irregular hypodense shadow. (**d**) Ground-glass appearance with an unclear edge and single or multiple “soap bubble” lucencies (indicated by the yellow arrow). (**e**) Osteolytic and polycystic changes involving a large part of the mandible with signs of teeth root displacement and enlargement of the involved region.

**Figure 3:**
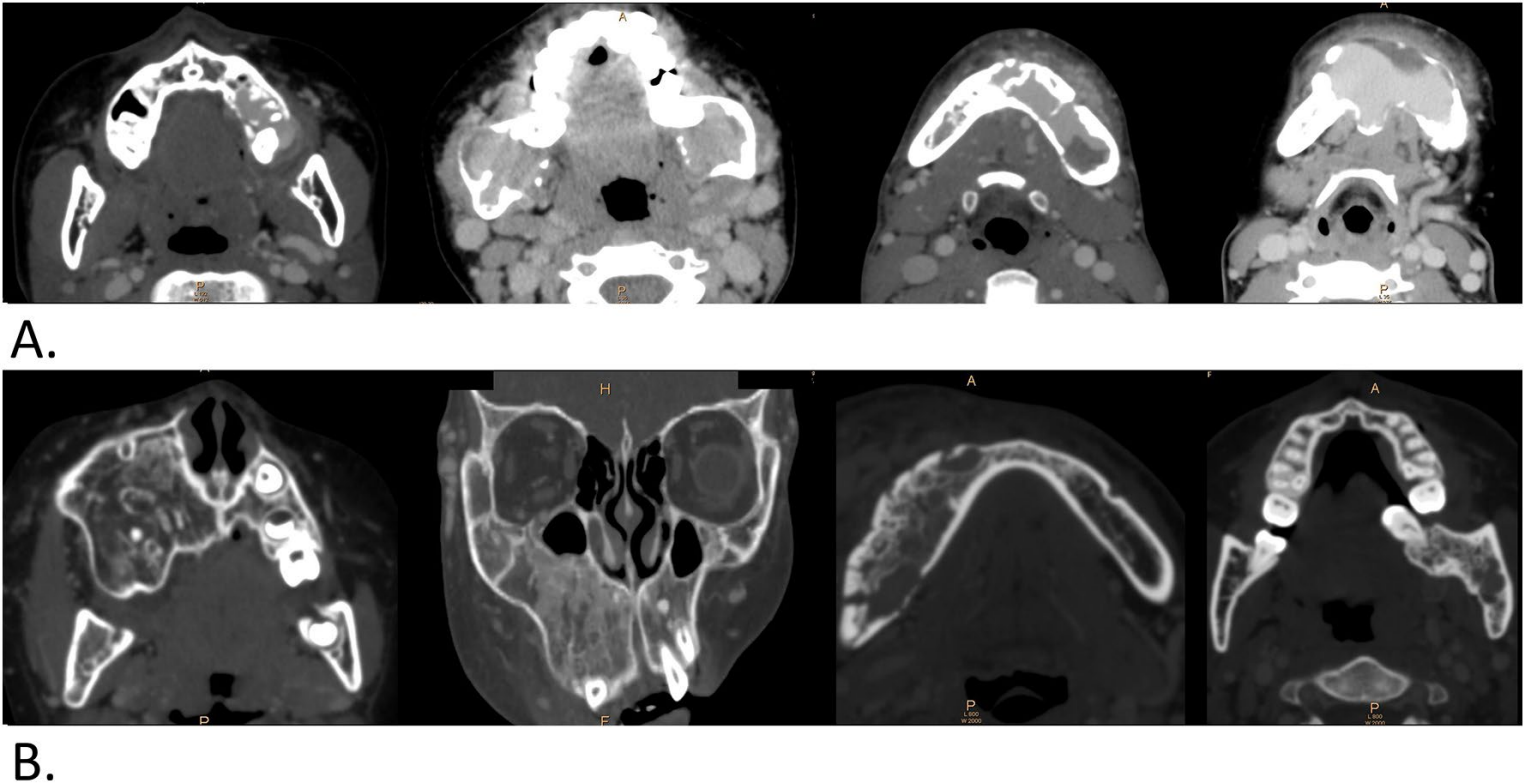
Enhancement in the cancellous bone on contrast-enhanced CT and manifestations of j-AVMs. Enhancement (**a**) centred on the root of the left first molar in the maxilla; the cancellous bone of the bilateral mandibular ramus; the anterior and posterior region of the mandible on the left side; the anterior region of the mandible with destruction and penetration of the cortical bone. (**b**) Irregular hyperosteogeny, with disordered and heterogeneous bone density and structure on contrast-enhanced CT and manifestations of j-AVMs. Irregular hyperosteogeny involves the whole maxilla of the left side; buccal cortex of the right mandible; area around the root of the teeth.

**Figure 4:**
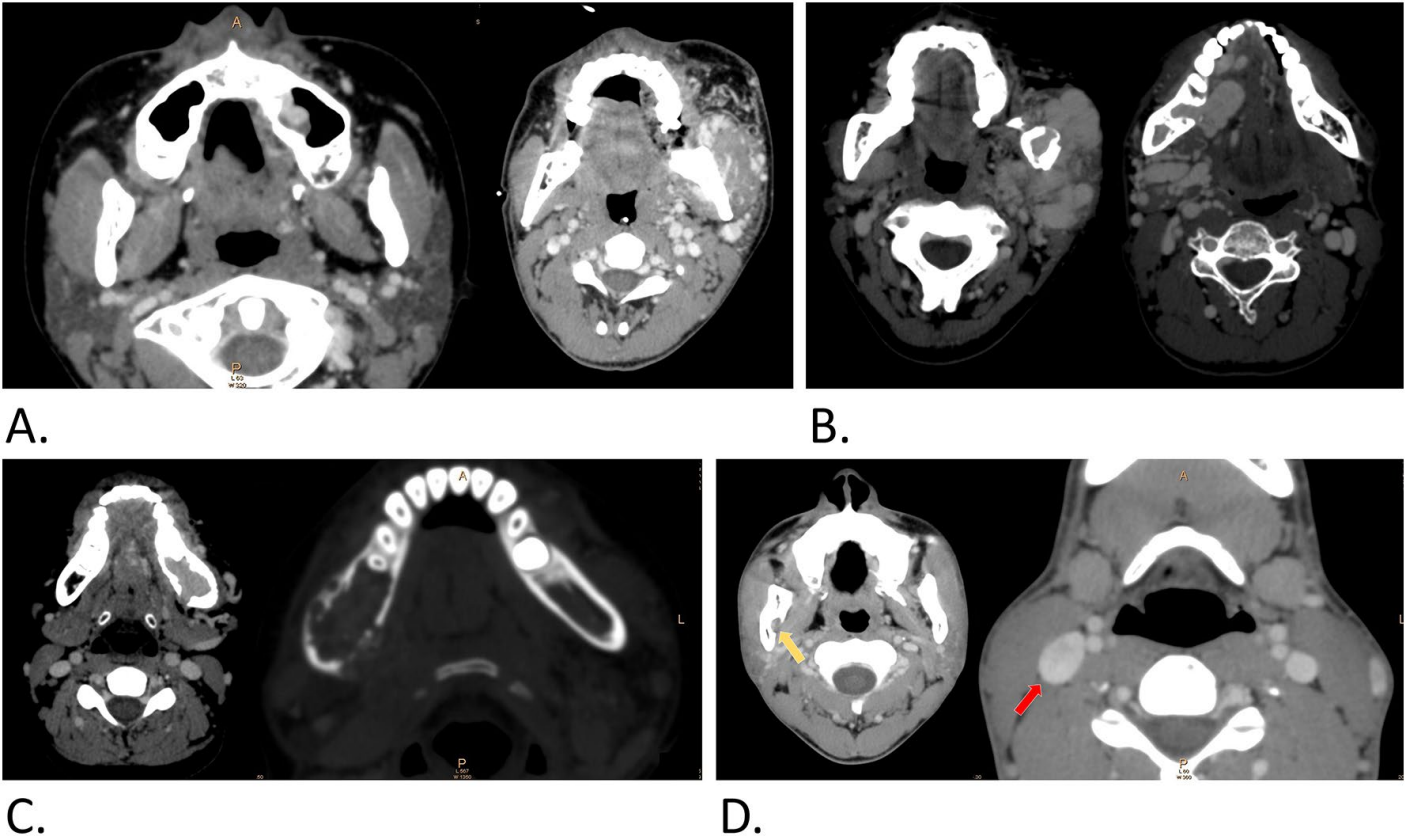
Other manifestations on contrast-enhanced CT of j-AVMs. (**a**) Involvement of the adjacent maxillary sinus and soft tissue. (**b**) Enlarged and tortuous draining veins showing abnormal arterial enhancement located inside or adjacent to either side of the jaw. (**c**) Signs of penetration of large vessels breaking through normally structured cortical bone. (**d**) Enlarged and wider mandibular foramen (yellow arrow) and enlarged external jugular vein (red arrow) compared with those on the contralateral side.

The j-AVMs presented different OPG manifestations in the maxilla and mandible. The j-AVMs in the maxilla presented on OPGs as an unclear maxillary sinus with a mild ground-glass appearance (Fig. 2a) and seldom as an obvious oval lucency. The manifestations presented on the OPGs of the j-AVMs in the mandible include (1) a clear oval or irregular lucency that is mostly centred on the root of the first molar with a long axis along the mandibular canal and occasionally with root resorption (Fig. 2b); (2) enlarged and wider mandibular canal relative to the contralateral side, and in some cases, the mandibular canal is replaced by an irregular hypodense shadow (Fig. 2c); (3) a ground-glass appearance with an unclear edge (Fig. 2d); (4) single or multiple “soap bubble” lucencies that are relatively small and distributed where the lesions are present, including the mandibular body and ramus (Fig. 2d); and (5) widely affected osteolytic and polycystic changes involving a large part of the mandible with signs of crushed and shifted teeth roots and enlargement of the involved region (Fig. 2e). One or more of these manifestations could be found in all cases.

### Contrast-enhanced CT manifestations

**Figure 5:**
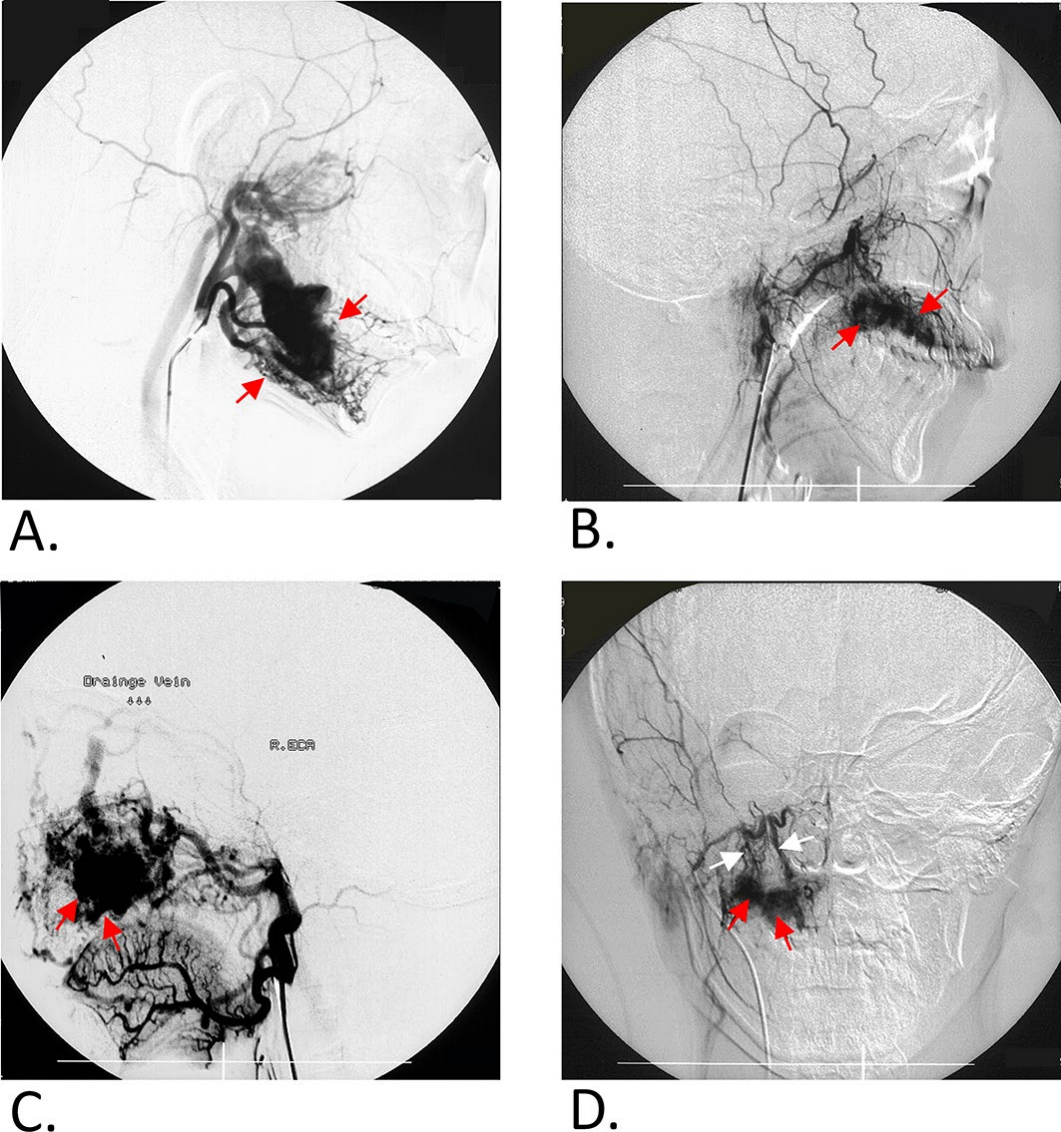
Digital subtraction angiography (DSA) showing abnormal arterial-venous shunts in patients with j-AVMs (**a**–**d**).

The j-AVMs lesions were evaluated by contrast-enhanced CT, which is timed from the arterial to venous phases. The maxillary and mandibular AVMs show consistent characteristics on contrast-enhanced CT. The J-AVM features presented on contrast-enhanced CT include (1) enhancement in the cancellous bone that is mostly centred on the root of the first molar and rarely in other parts of the jaw (Fig. 3a); (2) irregular hyperosteogeny with disordered and heterogeneous bone density and structure (Fig. 3b); (3) involvement of adjacent anatomic structures, such as the maxillary sinus and soft tissue (Fig. 4a); (4) enlarged and tortuous draining veins that show abnormal arterial enhancement located inside or adjacent to either side of the jaw (Fig. 4b); (5) penetration of large vessels breaking through normally structured cortical bone (Fig. 4c); (6) an enlarged and wider mandibular canal/foramen relative to the contralateral side (Fig. 4d); and (7) an enlarged external jugular vein relative to the contralateral side (Fig. 4d). One or more of these manifestations could be found in all cases. No pathological fractures were found in the present cohort.

## Discussion

J-AVMs are very rare conditions but pose a high risk of life-threatening haemorrhage. Aiming to develop effective management strategies for j-AVMs, recent studies have focused on the choice of various diagnostic modalities. However, as a type of “orphan” disease, j-AVMs have not been systematically assessed in previous studies because of their low incidence. Closely related to the oral cavity and teeth, j-AVMs have precise anatomical and physiological characteristics, which are different from AVMs in other parts of the body. As a result, it is challenging for clinicians to develop a comprehensive understanding and mastery of the clinical and imaging features of j-AVMs, which are of vital importance and serve as the cornerstone of the proper management of j-AVMs. The present study reviewed a cohort of 211 patients with j-AVMs in a single centre over a 15-year period and summarized the clinical and imaging features of j-AVMs, providing more evidence for the clear definition and diagnosis of j-AVMs. These finding can serve as the basis for future in-depth studies and can deepen our understanding of j-AVMs.

Based on the systematic review of clinical and imaging data in the present study, the following diagnostic criteria for j-AVMs were proposed by the interdisciplinary team: (1) an abnormal vascular varix with an arteriovenous fistula, dilated feeding arteries and earlier display of venous outflow in the arterial stage on angiography; (2) enhancement on contrast-enhanced computed tomography (CT) in the marrow of the jaw; and (3) potential clinical manifestations including extra-oral clinical signs (pulsation, higher skin temperature, and expanded superficial vein in the head and neck region) and intra-oral clinical signs (pulsation at the gingival groove, pulsatile loosening or floating teeth, and acute or chronic bleeding around the teeth).

Making the proper diagnosis is the cornerstone of managing patients with vascular malformations, according to Allan et al.^14^. Manifestations (characteristic arteriovenous shunt) on digital subtraction angiography (DSA) were widely accepted as the “gold standard” in the diagnosis of AVMs. However, an arteriovenous shunt also presents in certain neoplastic lesions^15^, which would lead to irreversible harm once misdiagnosed or if the diagnosis is delayed. Thus, it is insufficient to use a single detection method as a diagnostic criterion. Recently, it was also reported that different types of vascular malformations could be distinguished by multispectral optoacoustic tomography (MSOT)-based, non-invasive assessment of haemoglobin levels in vascular malformations^16^, which has not yet been extensively applied. The present study introduced comprehensive diagnosis criteria for j-AVMs, which were proposed and evaluated by a multidisciplinary team including oral and maxillofacial surgeons, dentists, radiologists and interventional radiologists. More attention was paid to the destruction of the local structure and function of the oral-maxillofacial region. Due to the superiority of bone imaging on CT and the relationship between lesions and bones in j-AVMs, the application of enhanced CT is recommended instead of magnetic resonance imaging (MRI). Manifestations of DSA have been reported as the “gold standard” in AVMs^14,17^, but arteriovenous shunts are also present in certain neoplastic lesions^15^. The present study emphasized the use of a combination of clinical signs and imaging manifestations as non-invasive pre-operative evaluations to describe the features of j-AVMs. The proposed diagnosis system considers the structure and function of the dental system in j-AVMs.

Because of the low incidence rate of j-AVMs, there are few studies on its prevalence and clinical characteristics. It is widely accepted that symptoms of AVMs often develop when the lesions grow large and cause haemodynamic disturbance, which often occurs during puberty and pregnancy^17^. Natural history can vary based on the location of the lesion, and there are few studies on the prevalence of j-AVMs with a relatively sufficient sample size, except for the report published by Joao Luiz and colleagues in 2018^11^, who performed a systematic literature review and analysis of mandibular AVMs that included 50 cases. The present study of 211 patients with j-AVMs who visited a medical centre in the past 14 years showed similar results to those previously reported. The authors found a mean age of 21.86 years and a median of 20 in patients with j-AVMs. Furthermore, the results showed a single peak at the age of 13 and a relatively higher plateau at the age range of 21–28, which indicated a potential relationship with changes in hormone levels in the body. Interestingly, the gender ratio showed a trend of more male than female patients with j-AVMs, with an approximate ratio of 4:3. This trend is consistent with results of previous studies and indicated that certain factors in the physiological environment of males might be associated with the occurrence or progression of j-AVMs, but further studies are needed for validation. Characteristic gender and age distributions of j-AVMs presented in this study provide epidemiological reference points for clinical diagnosis and management, which can reduce haemorrhage risks in cases of biopsy or resection performed without careful consideration.

As the symptomatology of AVMs is greatly variable, from a palpable thrill to haemorrhage or heart failure, depended on location and invasiveness of the lesion^11^, it is challenging but of vital importance for clinicians to clearly understand the unique clinical features of j-AVMs. The clinical course of j-AVMs, include being completely asymptomatic, having a swollen mass, bleeding, pain, growth disturbance, etc^14^. Of the 211 patients, 76.8% had bleeding as their chief complaint. Chronic gingival bleeding and life-threatening acute oral haemorrhage account for more than half of the chief complaints, which indicates the urgency and necessity of correctly understanding the clinical features of j-AVMs from the perspective of both dentists and radiological interventionists. It is noteworthy that 15.2% of the cohort in this study suffered from iatrogenic bleeding, which occurred in cases of hasty tooth extraction, local resection or biopsy without a clear preoperative diagnosis of j-AVMs. Such patients with iatrogenic bleeding were admitted to the emergency ward of the centre due to uncontrolled intraoperative haemorrhage. Interestingly, another circumstance of haemorrhage is nasal bleeding, which accounts for 7.6% of all complications of patients in the cohort. The anatomical basis of nose bleeding in patients with j-AVMs could possibly be related to the rich blood supply of the maxilla and pterygopalatine canal, which serves as an osseous channel between the oral and nasal cavities. Other symptoms were categorized into intraoral and extraoral symptoms, of which labial numbness was rare (2.8%) but possible in cases of damage or compression of the inferior alveolar nerve and often involves the lower lip and mental region. Notably, some patients with j-AVMs may present with a bruit, thrill, or pulsatility, which could be signs of less severe lesions in AVMs in other body parts. However, in j-AVMs, as the lesions are mainly in the bone marrow and limited by compact cortical bone, clinical manifestations may present insidiously. Milder clinical signs of j-AVMs do not reflect a smaller lesion, an earlier stage or a lower risk of haemorrhage. Attentions still need to be paid, and early intervention for j-AVMs is necessary.

Another interesting finding in the present study is the location-specificity of j-AVMs. J-AVMs in the mandible/left side/posterior regions are more frequent than those in the maxilla/right side/anterior regions (Table 1). Remarkably, the vast majority of j-AVMs were distributed in the posterior region (95.26%). The blood supply in cancellous bone of the posterior region is richer than that in the anterior region, which suggests a potential window revealing the pathological basic and pathogenesis of AVMs. However, it is contradictory that AVMs occur more frequently in the mandible than in the maxilla in the current cohort given that the maxilla has a more complex and abundant blood supply system. The exact cause of AVMs remains to be explored, although several possible theories of the pathogenesis of arteriovenous malformations have been proposed, including an embryologic theory, a local ischaemia theory, a theory of primitive artery-venous connection regression failure, etc^7^. Recently, progress has been made at the molecular and cellular levels related to the aetiology of AVMs, and somatic Kirsten rat sarcoma viral oncogene (KRAS)-activating mutations were found in AVMs in the brains^4,5^; however, more evidence is needed to explain the aetiology and location-specific features of j-AVMs. The location specificity of the occurrence of j-AVMs may allow for investigations of the potential causes of j-AVMs.

As one of the most important preoperative references, imaging manifestations of j-AVMs in the present cohort were variable. Although MRI and magnetic resonance angiography (MRA) are accepted as valuable modalities for detecting vascular anomalies and providing comprehensive assessments of lesions^14^, j-AVMs present unique imaging features with regard to the anatomy of the maxillofacial region and how the disease is managed. Despite a few drawbacks, OPGs play a role as a “first-line” diagnostic tool in certain circumstances before the identification of j-AVMs. Thus, a thorough knowledge of OPG manifestations of j-AVMs is of vital importance to make a suspected diagnosis of j-AVMs and to avoid invasive surgical procedures and consequent risk of life-threatening complications. In the present study, classic manifestations present in OPGs of j-AVMs were identified separately, depending on whether they were in the maxilla or mandible, as marked differences were noted. J-AVMs of the mandible present with distinct characteristics in OPGs, providing valuable and strong refence information for a definite diagnosis and for clarifying the relationship between teeth and the lesion. In contrast, j-AVMs of the maxilla might present with only an unclear maxillary sinus or nearly normal imaging in some cases, and a comprehensive analysis based on clinical manifestations such as pulsatile mass/pulsatile floating teeth/bleeding in the oral or nasal cavity in necessary. Upon a suspected diagnosis of j-AVMs, the application of enhanced CT is recommended based on the superiority of bone imaging of this method and the strong relationship between lesions and jaws. Classic j-AVM features in contrast-enhanced CT were identified in the present study, of which enlarged and tortuous draining veins are of vital importance in the diagnosis of j-AVMs. Previous studies have described several features of j-AVMs on enhanced CTs as case series with a small sample size (12). The present study reviewed the manifestations of enhanced CTs and OPGs of 211 patients with j-AVMs, and variable manifestations were concluded. One of the interesting findings is that j-AVMs in the mandible occasionally present ground-glass appearance and might cause confusion and lead to misdiagnosis by clinicians and invasive biopsies without careful consideration, which could result in emergencies and even death. Contrast-enhanced CT and digital subtraction angiography are strongly recommended under these circumstances. We speculated that sporadic lucency was a result of the outflow vein of j-AVMs penetrating through cortex bone, but further evidence is needed. Another interesting finding is that the endosseous scalloping of the cortex was shown to be associated with the wide osteolytic and polycystic changes involving a large part of the mandible, which might be related to the enlargement of the vascular malformations.

The retrospective study design is a potential limitation of this study. However, as the prevalence of j-AVMs is low, it is hard to collect a convincingly large and uniform sample group in several years; however, a prospective study is still needed to better evaluate the clinical and imaging feature of j-AVMs. Another possible limitation is the absence of pathological evidence, which have introduced selection bias. However, as AVMs present with a high tendency of uncontrolled haemorrhage, incisional biopsy is extremely dangerous. In addition, during the 1–15-year follow-up period, the medical histories of the patients all supported a diagnosis of j-AVMs.

In summary, j-AVMs, previously known as central haemangioma of the jaw are very rare congenital high-flow vascular anomalies with a relatively high tendency of haemorrhage and unique clinical and imaging features. A comprehensive diagnose system based on clinical and imaging features of j-AVMs could provide valuable reference data for clinical management of j-AVMs and help avoid improper iatrogenic trauma or delayed treatment.

## Methods

### Patients

Approval was obtained from the institutional review board of the hospital for a retrospective review of patient medical and imaging records. Informed consent has been obtained from a parent and/or legal guardian of every underage participant. Informed consent for publication of identifying information/images in an online open-access publication was also obtained. The study conforms to STROBE Guidelines. The inclusion criteria were as follows: (1) patients at Shanghai Ninth People’s Hospital, Shanghai Jiao Tong University School of Medicine, treated between November 2003 and November 2017 with digital subtraction angiography (DSA) showing abnormal arterial-venous shunts (Fig. 5) and (2) patients agreeing to participate. The exclusion criteria were as follows: (1) patients who did not provide informed consent and (2) patients with incomplete information (clinical records, imaging data, etc.). In total, 211 patients with j-AVMs were enrolled in this analysis, including 120 males (mean age 22.07, age range 4–61) and 91 females (mean age 21.89, age range 3–57). 42 patients in this series have been reported separately in previous studies^3,18–20^ as case series with a maximum sample size of 18 cases in a single report without a systematic analysis. A large sample size of j-AVMs (211) during a period of 15 years was included and classic features of j-AVMs were analysed and concluded by a multidisciplinary team in the present study.

### Data collection

All medical records and databases were reviewed by an interdisciplinary team (including two interventional radiologists with 15 years of experience each, one radiologist with 8 years of experience, one oral and maxillofacial surgeon with 10 years of experience and three dentists with 5 years of experience each). The data collected included demographics, presentation signs and symptoms, and manifestations on enhanced CT and orthopantomograms (OPGs). Teeth involvement was evaluated in the anterior region (incisors and canines) and posterior region (premolars and molars). Histopathologic confirmation was not required for diagnosis and was intentionally avoided because of the high risk of haemorrhagic shock in biopsy operations.

### Radiological examination techniques

The anatomical and imaging characteristics were evaluated through radiological examinations, including orthopantomograms (OPGs), contrast-enhanced CT and angiography. OPGs were used for the initial evaluations of the j-AVM lesions. Contrast-enhanced CT was performed with a 64-channel multidetector CT unit including both thin-section axial images and multiplanar reconstructed images at a thickness of 1.25 mm to obtain detailed anatomic and haemodynamic information. The OPG and contrast-enhanced CT images were reviewed and comprehensively analysed by the interdisciplinary team to confirm the diagnosis, define the location and observe the angioarchitecture. Angiograms of the related artery, namely, the external carotid artery, were also reviewed by the interdisciplinary team.

### Statistical analysis

GraphPad 8.0 software (Inc., CA, USA) was applied for the data analysis and visualization.

### Institutional review board statement

The study was performed in accordance with the declaration of Helsinki and experimental protocols was revised and approved by Scientific Research Projects Approval Determination of Independent Ethics Committee of Shanghai Ninth People’s Hospital affiliated with Shanghai Jiao Tong University, School of Medicine, No. 2017070.

### Informed consent statement

Informed consent was obtained from all individual participants included.

## Data Availability

All relevant data are available from the corresponding author upon reasonable request.
